# Short Hairpin RNA Silencing of PHD-2 Improves Neovascularization and Functional Outcomes in Diabetic Wounds and Ischemic Limbs

**DOI:** 10.1371/journal.pone.0150927

**Published:** 2016-03-11

**Authors:** Kevin J. Paik, Zeshaan N. Maan, Elizabeth R. Zielins, Dominik Duscher, Alexander J. Whittam, Shane D. Morrison, Elizabeth A. Brett, Ryan C. Ransom, Michael S. Hu, Joseph C. Wu, Geoffrey C. Gurtner, Michael T. Longaker, Derrick C. Wan

**Affiliations:** 1 Hagey Laboratory for Pediatric Regenerative Medicine, Department of Surgery, Plastic and Reconstructive Surgery Division, Stanford University School of Medicine, Stanford, CA, United States of America; 2 Department of Medicine, Division of Cardiology, Stanford University School of Medicine, Stanford, CA, United States of America; 3 Institute for Stem Cell Biology and Regenerative Medicine, Stanford University School of Medicine, Stanford, CA, United States of America; University of Illinois at Chicago, UNITED STATES

## Abstract

The transcription factor hypoxia-inducible factor 1-alpha (HIF-1α) is responsible for the downstream expression of over 60 genes that regulate cell survival and metabolism in hypoxic conditions as well as those that enhance angiogenesis to alleviate hypoxia. However, under normoxic conditions, HIF-1α is hydroxylated by prolyl hydroxylase 2, and subsequently degraded, with a biological half-life of less than five minutes. Here we investigated the therapeutic potential of inhibiting HIF-1α degradation through short hairpin RNA silencing of PHD-2 in the setting of diabetic wounds and limb ischemia. Treatment of diabetic mouse fibroblasts with shPHD-2 *in vitro* resulted in decreased levels of PHD-2 transcript demonstrated by qRT-PCR, higher levels of HIF-1α as measured by western blot, and higher expression of the downstream angiogenic genes SDF-1 and VEGFα, as measured by qRT-PCR. *In vivo*, shPHD-2 accelerated healing of full thickness excisional wounds in diabetic mice compared to shScr control, (14.33 ± 0.45 days vs. 19 ± 0.33 days) and was associated with an increased vascular density. Delivery of shPHD-2 also resulted in improved perfusion of ischemic hind limbs compared to shScr, prevention of distal digit tip necrosis, and increased survival of muscle tissue. Knockdown of PHD-2 through shRNA treatment has the potential to stimulate angiogenesis through overexpression of HIF-1α and upregulation of pro-angiogenic genes downstream of HIF-1α, and may represent a viable, non-viral approach to gene therapy for ischemia related applications.

## Introduction

Chronic wounds, often associated with peripheral limb ischemia in diabetic and aged individuals [1], severely impair quality of life and exert a substantial burden on health care systems worldwide, with an estimated 6.5 million patients with chronic wounds managed each year in the United States alone [2]. An impaired response to hypoxia is a major factor contributing to compromised wound healing [3]. Hypoxia inducible factor-1 (HIF-1) regulates the majority of adaptive cellular responses to hypoxia, and consists of a highly regulated α-subunit and a constitutively expressed β-subunit [3]. Under hypoxic conditions the hydroxylation of proline residues on the HIF-1α subunit by prolyl hydroxylases (PHDs) is inhibited, which blocks the degradation of HIF-1α [4]. HIF-1α stabilization promotes pro-angiogenic gene transcription, stimulating neovascularization. Under normoxic conditions, however, HIF-1α is hydroxylated by Prolyl Hydroxylase Domain-2 (PHD-2), ubiquitinated, and eventually degraded [5, 6].

Current therapies for chronic wounds include topical growth factor monotherapy, which, though promising in animal studies, has not yet found its way into regular clinical practice [7]. Stem cell based therapy, thought to support neovascularization through the secretion of growth factors, may be another promising approach to treating chronic wounds. Unfortunately, recent evidence shows that stem cell function is also susceptible to biological aging and is negatively impacted by diabetes [8], diminishing the regenerative potential of autologous treatments in those patients most in need [9, 10]. We and others have shown that upregulation of HIF-1α improves wound healing by increasing the expression of angiogenic factors [3, 11, 12]. A reduction of prolyl hydroxylase activity has been shown to stabilize HIF-1α, induce hypoxia-inducible genes, and stimulate angiogenesis [13, 14]. Furthermore, knockout of PHD-2 leads to the inhibition of HIF-1α degradation, stimulating angiogenesis and accelerating healing [15].

Here we describe the generation and use of a short hairpin RNA (shRNA) designed to target and knockdown PHD-2 expression, with the aim to stabilize endogenous levels of HIF-1α protein through inhibition of its degradation. We found that in diabetic mouse fibroblasts *in vitro*, shRNA knockdown of PHD-2 transcript resulted in higher levels of HIF-1α protein, as well as upregulation of downstream, angiogenic transcripts SDF-1 and VEGFα. We then tested the efficacy of PHD-2 knockdown *in vivo* by injecting shPHD-2 into full thickness excisional wounds on the dorsa of diabetic mice and saw higher levels of HIF-1α protein and upregulation of the angiogenic transcript PDGFα in wound beds, as well as enhanced staining for CD31 and accelerated wound healing overall. Finally, we delivered shPHD-2 to the ischemic hind limbs of aged mice, observing enhanced perfusion and muscle bulk survival as a result.

## Materials and Methods

### Ethics Statement

All mice were housed in the Stanford University Veterinary Service Center in accordance with NIH and Stanford University Institutional Animal Care and Use Committee guidelines. This study and its experiments were approved and performed in accordance with the Stanford University Institutional Animal Care and Use Committee (APLAC protocols #21308 and #12080).

### Construction of shPHD-2 Plasmid

**Fig 1:**
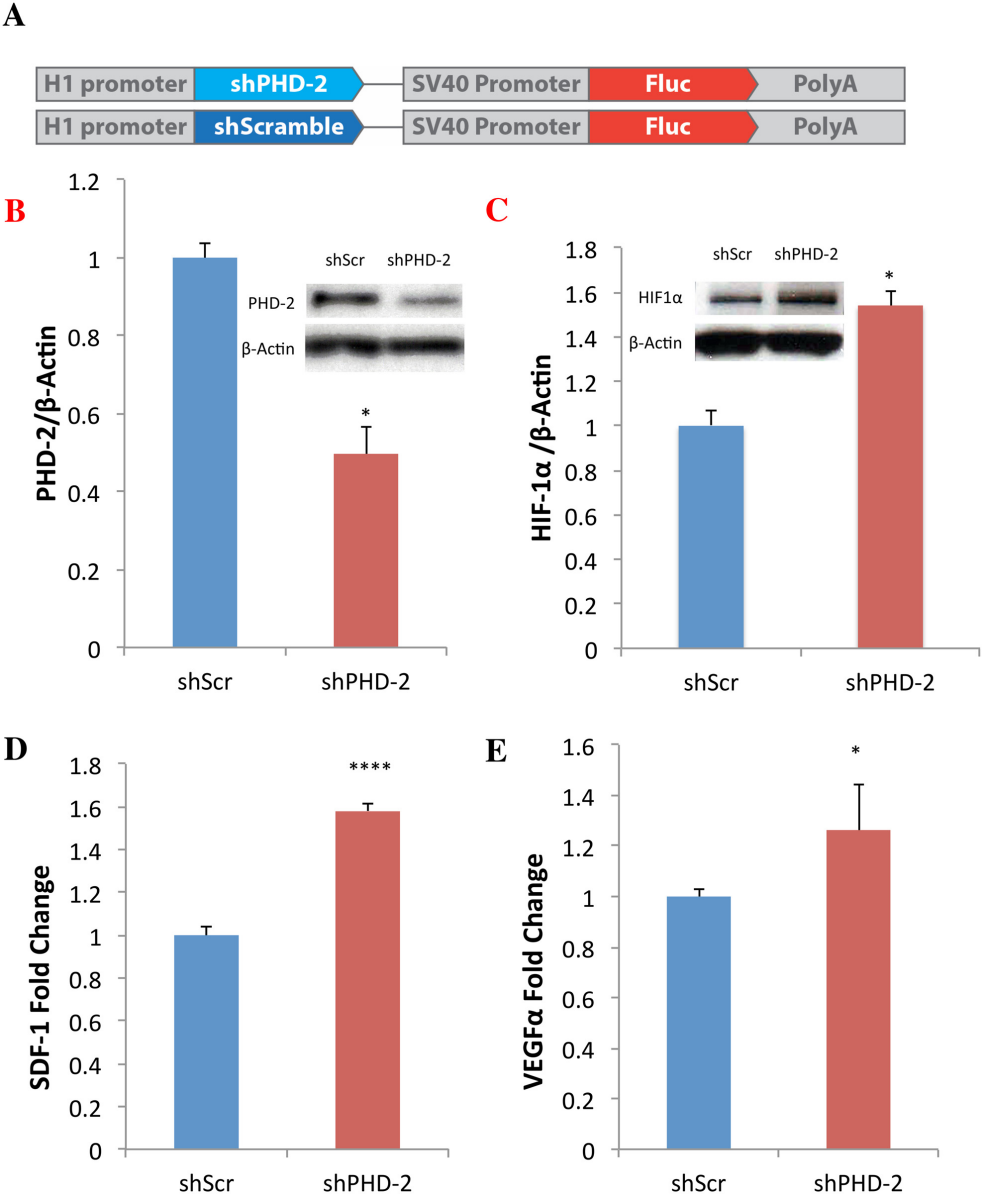
shPHD-2 Suppresses PHD-2 and Upregulates HIF-1α and Downstream Angiogenic Genes In Vitro. Cells for all experiments were cultured under hypoxic conditions of 5% oxygen. (a) Schematics of shPHD-2 and shScr constructs. (b) qRT-PCR showed decreased PHD-2 expression *in vitro* from diabetic mouse fibroblasts transfected with shPHD-2 compared to fibroblasts transfected with shScr (****p<0.0001). (c) Western blot revealed higher levels of HIF-1α protein from shPHD-2 fibroblasts compared to shScr fibroblasts. (d) qRT-PCR additionally showed that SDF-1, an angiogenic chemokine downstream of HIF-1α, was more highly expressed in the shPHD-2 group than in shScr *in vitro* (****p<0.0001), as was (e) VEGFα (*p<0.05).

pGL3–Control vector (Promega, Madison, WI) was used as the backbone for construction of the shRNA vector. Briefly, an H1 promoter with 3’–flanking XbaI and XhoI restriction sites was amplified with 5’-KpnI and 3’-NheI restriction sites from the pFHUUIG shortU6 vector obtained as a gift from the Südof lab (Stanford University, Stanford, CA). shRNAs targeting PHD-2 (shPHD2_2, shPHD2_3, and shPHD2_A) and a scramble shRNA (shScr) were designed using previously validated shRNAs sequences (14, 16), and contained overhangs complementary to 3’-XhoI and 5’-XbaI (Fig 1a). The shRNA oligos (IDT, Berkeley, CA) were allowed to anneal after combining equimolar concentrations of each complementary strand, heating to 95°C in a hot water bath for five minutes, and allowing to cool to room temperature over four hours. The oligos were then phosphorylated with T4 polynucleotide kinase (NEB, Ipswich, MA). The H1 promoter and shRNA sequences were inserted sequentially. The final vector sequence was confirmed with sequencing (PAN Facility).

### Harvesting and Culture of Fibroblasts

Fibroblasts were harvested from 6-week-old, female diabetic Lepr db/db mice (The Jackson Laboratory, Bar Harbor, Maine), using dispase and collagenase (Sigma Aldrich). Harvested cells were cultured at 37°C in 6-well plates containing fibroblast culture medium [Dulbecco’s Modified Eagle Medium (DMEM), 10% Fetal Calf Serum (FCS), 1% MEM Non-essential Amino Acid Solution, 1% penicillin/streptomycin]. Each well was either transfected with 2 μg of shPHD-2 plasmid (equal parts of shPHD2_2, shPHD2_3, and shPHD2_A) or 2 μg of shScr plasmid using FuGENE (Promega) according to the manufacturer’s protocol and cultured in a hypoxic chamber maintained at 5% oxygen. After 24 hours, samples from each group were collected in Trizol (Invitrogen, Carlsbad, CA) and RIPA buffer [50 mmol/L of pH 7.5 HEPES, 150 mmol/L of NaCl, 1 mmol of EDTA, 10% glycerol, 1% Triton-X-100, 25 mM sodium fluoride] containing 1 mM sodium orthovanadate and Proteases Inhibitor Cocktail (Sigma Aldrich, St. Louis, MO) for RNA and protein analysis respectively.

### Reverse Transcription and qRT-PCR (in vitro and in vivo)

RNA from *in vitro* fibroblast samples and *in vivo* tissue samples was collected then phase separated using Trizol (Invitrogen) and isolated using the RNeasy Mini Kit (Qiagen). Isolated RNA was quantified using Nanodrop 2000 (Thermo Fisher Scientific, Waltham, MA) then reverse-transcribed using TaqMan Reverse Transcription Reagents (Invitrogen). cDNA was examined using quantitative real-time PCR (qRT-PCR) with the Applied Biosystems Prism 7900HT sequence detection system (Applied Biosystems, Foster City, CA) and Power SYBR Green PCR Master Mix (Applied Biosystems). Target quantities were normalized to endogenous β-actin quantities using the standard curve method. Normalized quantities were then calibrated to baseline expression of shScr to generate relative expression levels. Primer sequences were obtained from PrimerBank (S1 Table).

### Western Blot (in vitro and in vivo)

*In vitro* fibroblast samples and *in vivo* tissue samples were lysated with cold RIPA buffer, sonicated, and quantified using a BCA Assay (Thermo Fisher Scientific). Cell lysates (50 μg) were electrophoresed on a NuPage 4–12% Bis-Tris gel (Novex, Carlsbad, CA) and transferred to a PVDF membrane, which was blocked in 5% non-fat milk for one hour. Immunoblotting was performed using a primary rabbit anti-mouse HIF-1α antibody (Santa Cruz Biotechnology, Santa Cruz, CA) at a 1:400 dilution at 4°C overnight. Following the primary antibody incubation, the membrane was incubated with horseradish-peroxidase conjugated secondary anti-rabbit antibody (Cell Signaling) at a 1:3000 dilution for one hour. Chemiluminescence was detected using the Amersham ECL Western Blotting Detection Reagents (GE Healthcare, Little Chalfont, UK). Densitometry analysis of electrophoretic bands was performed using ImageJ (NIH, Bethesda, MD). The density of HIF-1α bands was normalized to endogenous β-actin (Cell Signaling), and presented as percentage increase.

### Full Thickness Excisional Wound Model

Full thickness 6 mm excisional wounds were made on the dorsa of 6-week-old, female diabetic Lepr db/db mice (The Jackson Laboratory; n = 5 mice per group, for n = 10 wounds per group) as previously described [16]. 100 μg of shPHD-2 or shScr plasmid was added to each wound, divided into four 25 μg fractions and injected into the dermal layer of each wound at 12 o’clock, 3 o’clock, 6 o’clock, and 9 o’clock positions. After five days, two mice per group (n = 4 wounds per group) were sacrificed and tissue surrounding the wounds was harvested and placed in Trizol (Invitrogen) and RIPA buffer for RNA and protein analysis, respectively. The remaining mice (n = 3 mice per group, for n = 6 wounds per group) were monitored and wounds were photographed every two days until wound closure. Upon closure, wounds were embedded and frozen in OCT (Sakura Finetek USA, Inc., Torrance, CA) for histology.

### Photometric Wound Healing Analysis

Photometric analysis of wounds was carried out in Adobe Photoshop CS6 (Adobe, San Jose, CA). For each wound, the magnetic lasso tool was used to measure the area of the wet wound, which was then normalized to the area measured within the surrounding silicone ring. Normalized wound sizes were then calibrated to baseline wound size (100%).

### Histologic Quantification of CD31 Staining

Healed wounds were harvested, bisected, and fixed in 4% paraformaldehyde for 12 hours at 4°C. Fixed tissue was dehydrated and embedded in paraffin blocks. 8-μm-thick sections were serially cut and incubated with a polyclonal rabbit anti-mouse anti-CD31 primary antibody (1:100, Abcam, Cambridge, UK) overnight at 4°C, followed by Alexa Fluor 594 Goat Anti-Rabbit IgG secondary at room temperature (1:200, Invitrogen) for one hour. All samples were counterstained with DAPI. Slides were mounted with the Vectashield Mounting Medium (Vector Laboratories, Burlingame, CA) and cover-slipped. A Zeiss Axioplan 2 fluorescence microscope was used to image the slides (Carl Zeiss, Inc., Thornwood, NY). Quantification of fluorescence was performed by a blinded observer analyzing at least five high-powered fields per wound at 200x using ImageJ software (NIH) as previously described [17].

### Unilateral Hind Limb Ischemia Model

Unilateral hind limb ischemia was created in 18-month-old female wild-type C57BL/6J mice (National Institute on Aging, Bethesda, MD) as described previously (n = 4) [18, 19]. Briefly, mice were anesthetized with inhalational anesthetic and placed in a supine position. The right inguinal area was cleaned, depilated, and prepped for surgery and the right femoral artery was dissected free along its entire length (from the inguinal ligament to the popliteal artery) under an operating microscope. Paired double knots of 7–0 silk suture were used to occlude the femoral artery proximally and distally, all branches were ligated, and the entire artery was sharply excised. The skin incision was closed with 5–0 Vicryl sutures. Immediately following surgery, ischemic limbs were treated with either shScr or shPHD-2 plasmid via intra-gastrocnemius injection of 300 μg of DNA in 20 μl of sterile saline using a 31-gauge needle. The left hind limb was kept intact and used as a non-ischemic limb control. Ischemic revascularization was tracked via laser doppler blood perfusion on Days 0, 4, 10 and 14 following injury. At Day 14, animals were sacrificed and ischemic and control limbs were harvested for histologic quantification of muscle survival.

### Laser Doppler Blood Perfusion

Mice were anesthetized with inhalational anesthetic and the bilateral inguinal area was cleaned and depilated. Animals were then placed on a 37°C heated surface for five minutes under continuous flow of isoflurane, with core temperature monitored to ensure euthermia. Each animal was then placed in a supine position on black pad for laser doppler imaging of the bilateral hind limbs using a PeriScan PIM3 laser doppler system (Perimed AB, Stockholm, Sweden). Regions of interest of the ischemic or non-ischemic hind limb were drawn in a standard fashion. Perfusion in the ischemic and normal limbs was quantified using the mean pixel value within the region of interest, and the relative changes in hind limb blood flow were expressed as the ratio of the right (ischemic) over left (normal) mean pixel value [18, 20].

### Histologic Quantification of Muscle Survival

Muscle survival within the ischemic hind limbs was assessed utilizing hematoxylin and eosin (H&E) histological examination (n = 4). Briefly, the gastrocnemius muscle from ischemic and control hind limbs was harvested and immediately embedded in OCT. H&E immunohistochemical staining of 7-μm-thick sections was used to assess muscle survival based on normal muscle tissue architecture (shown as Control) as compared to fibrotic and necrotic tissue. Intensity threshold values were set automatically and quantification of eosin stained muscle was determined by pixel-positive area per high power field (ImageJ). For each limb, four high-powered fields at 200x were recorded and averaged.

### Statistical Analysis

Numerical data are presented as means ± standard error. In figures, bar graphs represent means, and error bars represent standard error. Unless otherwise stated, statistical analysis was performed using a one way analysis of variance (ANOVA) for multiple group comparisons and a two-tailed Student’s *t-*test was used to directly compare two groups. A value of *p < 0.05 was considered significant.

## Results

### shPHD-2 Suppresses PHD-2 and Upregulates HIF-1α and Downstream Angiogenic Genes In Vitro

We first examined the effects of shPHD-2 therapy by transfecting diabetic mouse fibroblasts *in vitro* with either shPHD-2 or shScr plasmids. shScr plasmids were used as controls to ensure that any phenotype observed was not the result of off-target sh plasmid effects. Total RNA was collected from transfected fibroblasts after allowing 24 hours for recovery. qRT-PCR analysis demonstrated that diabetic mouse fibroblasts transfected with shPHD-2 resulted in significantly decreased levels of PHD-2 transcript, compared to fibroblasts transfected with the shScr control (****p<0.0001) (Fig 1b). Protein was also collected from the transfected diabetic mouse fibroblast samples. Western blot analysis congruously showed higher levels of HIF-1 present in fibroblasts treated with shPHD-2 compared to fibroblasts transfected with shScr (Fig 1c). Transcriptional analysis from qRT-PCR additionally revealed that angiogenic genes downstream of HIF-1, SDF-1 (****p<0.0001) and VEGF (*p<0.05), were upregulated among diabetic mouse fibroblasts transfected with shPHD-2 compared to shScr control (Fig 1d and 1e).

### shPHD-2 Promotes Angiogenesis and Accelerated Wound Healing In Vivo

**Fig 2:**
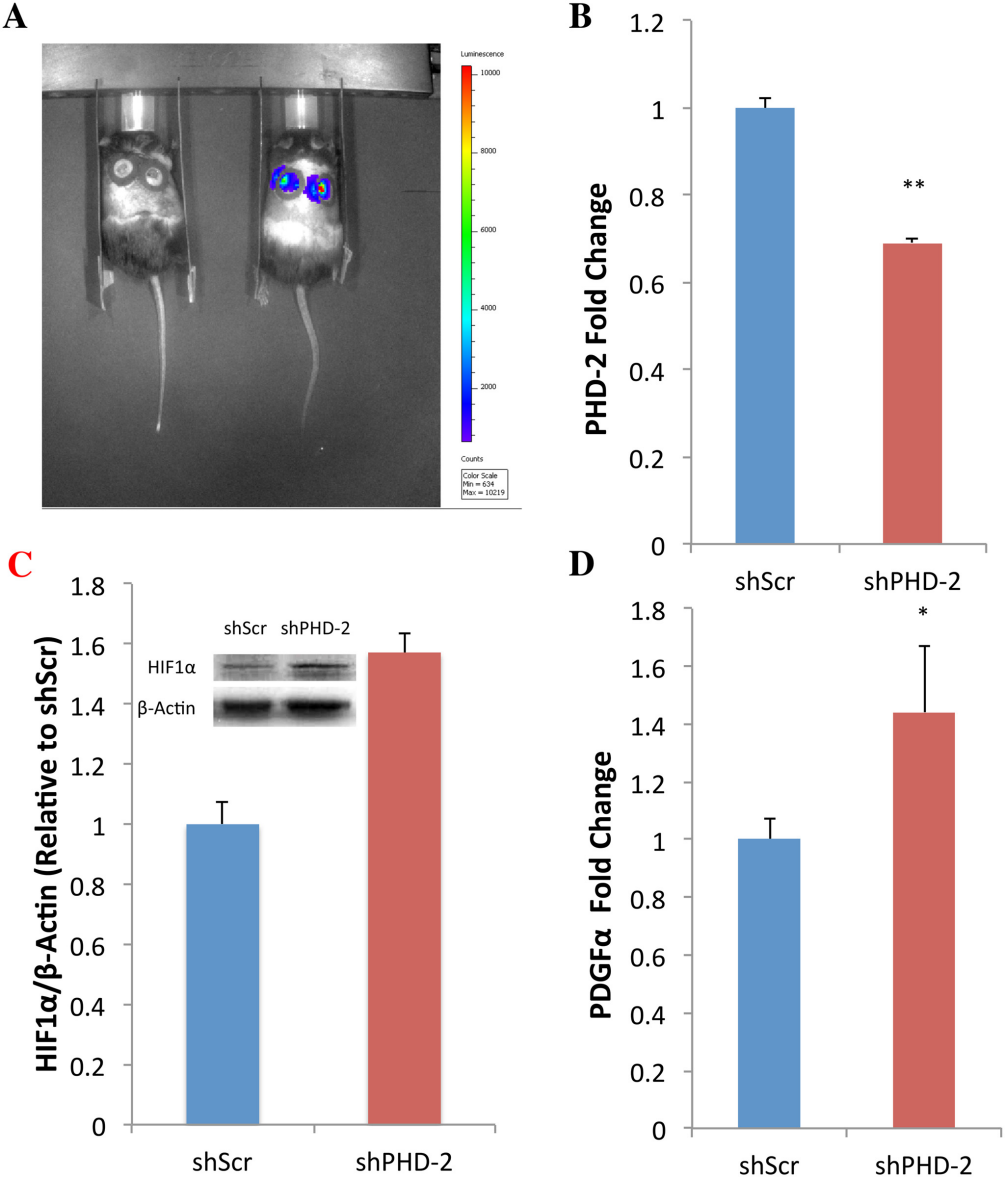
shPHD-2 Promotes Angiogenesis *In Vivo*. (a) BLI confirmed uptake of shPHD-2 plasmid *in vivo* as indicated by expression of firefly luciferase (right) compared to a non-injected mouse (left). (b) RNA derived from the tissue surrounding the wound beds five days after injection showed decreased PHD-2 transcript as a result of shPHD-2 plasmid treatment compared to shScr (**p<0.01). (c) Protein derived from the same tissue samples congruously showed higher levels of HIFα protein in the shPHD-2 group. (d) qRT-PCR further demonstrated the upregulation of angiogenic gene PDGFα in the shPHD-2 treatment group (*p<0.05).

**Fig 3:**
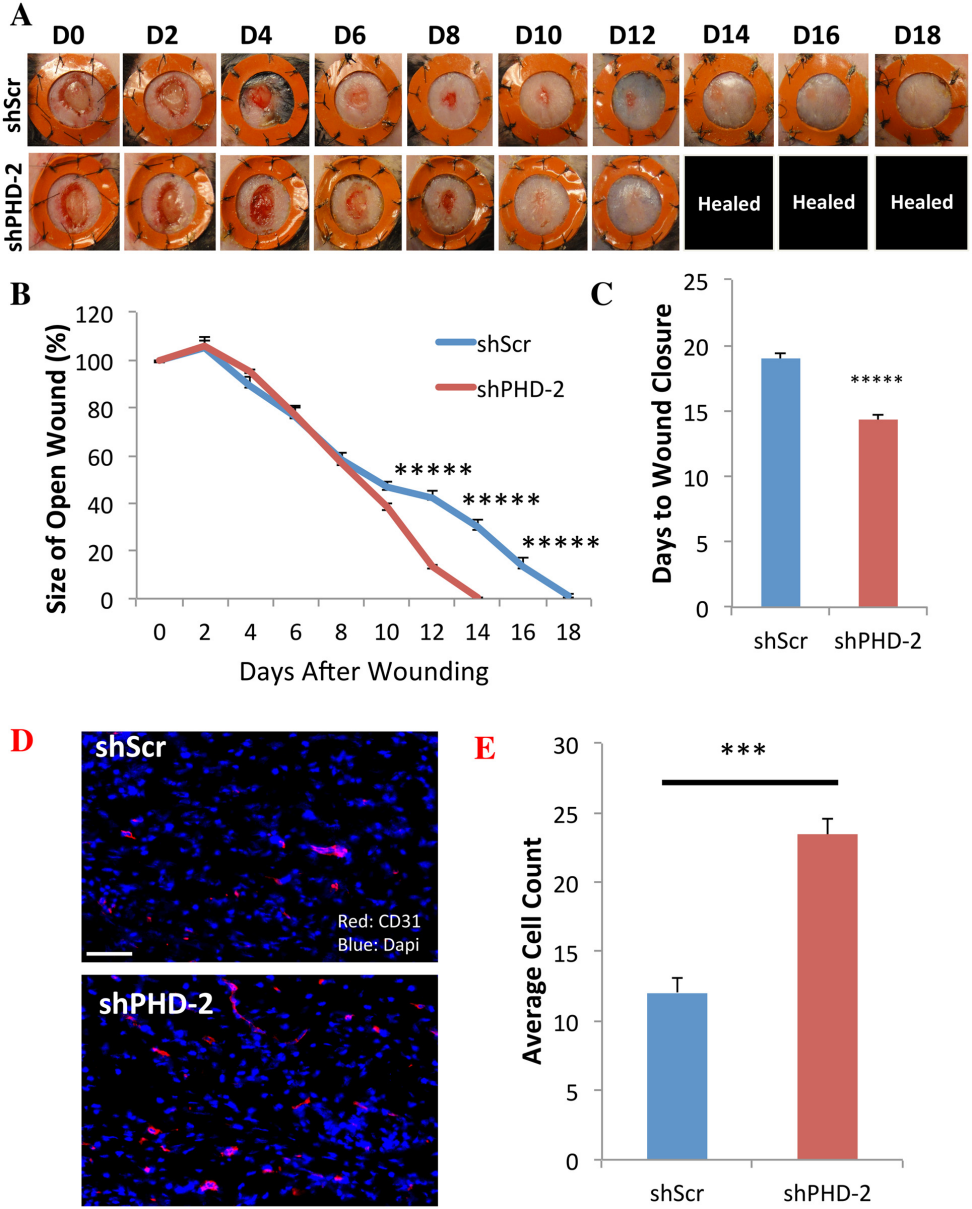
shPHD-2 Promotes Accelerated Wound Healing and Increased Vascular Density *In Vivo*. (a,b) Diabetic wounds treated with shPHD-2 healed significantly faster than wounds treated with shScr, (c) closing in an average of 14.33 ± 0.45 days compared to 19 ± 0.33 days (*****p<0.00001). (d,e) CD31 staining revealed enhanced vascular density in wound beds treated with shPHD-2 versus shScr (***p<0.001). Scale bar = 100μm.

To evaluate shPHD-2 therapy *in vivo*, 6 mm full thickness excisional wounds were created on the dorsa of diabetic mice and injected with either shPHD-2 or shScr plasmid. *In vivo* transfection was confirmed through the use of bioluminescence imaging (BLI), which allowed firefly luciferase expression from the plasmid constructs to be detected in the treated wound beds (Fig 2a). qRT-PCR analysis of the RNA gathered from the tissue surrounding the wound beds five days after initial injection, confirmed decreased expression of PHD-2 transcript *in vivo* as the result of shPHD-2 treatment (**p<0.01) (Fig 2b). Western blot analysis using protein from the same *in vivo* samples in turn showed increased HIF-1 protein in shPHD-2 wound beds compared to shScr (Fig 2c). And qRT-PCR also revealed higher downstream expression of angiogenic gene PDGF in the shPHD-2 group compared to shScr (*p<0.05) (Fig 2d). Overall, when shPHD-2 was injected into the wound beds of diabetic mice, wound healing was accelerated compared to the shScr control group (Fig 3a and 3b). shPHD-2-treated wounds were observed to close within 14.33 ± 0.45 days of injury while shScr-treated wounds closed at 19 ± 0.33 days (*****p<0.00001) (Fig 3c). Histological analysis confirmed an increased vessel count in the healed shPHD-2 wound beds compared to the shScr wound beds at the time of sacrifice (***p<0.001) (Fig 3d and 3e).

### Local Injection of shPHD-2 Accelerates Revascularization Following Ischemic Injury and Improves Tissue Survival

**Fig 4:**
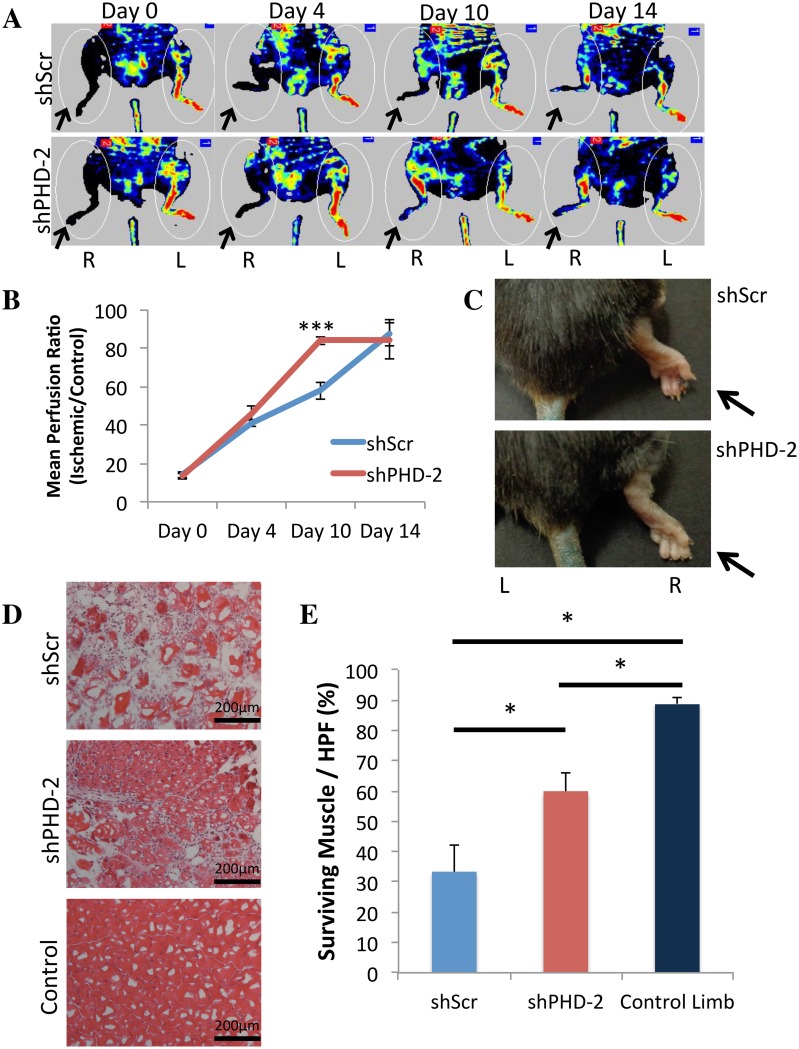
Local Injection of shPHD-2 Accelerates Revascularization Following Ischemic Injury and Improves Tissue Survival. (a) Representative laser doppler images of ischemic and control limbs for shPHD-2 and shScr-treated animals demonstrating accelerated neovascularization with shPHD-2 application (arrows indicate ischemic limbs). (b) Quantification of mean perfusion of ischemic to control limbs, with the greatest difference between shPHD-2 (red) and shScr (blue) occurring at Day 10 (***p<0.001). (c) Distal toe necrosis was observed in the shScr group, but not in shPHD-2 treated limbs (arrows indicate ischemic limbs). (d) Representative high-power H&E stained images (200x) and (e) quantification revealing enhanced muscle survival at Day 14 in ischemic hind limbs treated with shPHD-2 as compared to shScr (*p<0.05).

To assess the effect of shPHD-2 on neovascularization following ischemic injury, a validated murine model of hind limb ischemia was utilized. Consistent with the pro-vascular effect seen in diabetic wounds, application of shPHD-2 to aged ischemic limbs significantly accelerated revascularization (mean perfusion ratio on Day 10 post injury of 84.0 ± 2.2% for shPHD-2 treated versus 57.7 ± 4.3% for shScr controls, ***p<0.001) (Fig 4a and 4b). Additionally, shPHD-2 delivery promoted tissue survival within ischemic limbs, with an absence of distal toe necrosis observed in shPHD-2 treated mice (Fig 4c). Significantly greater neovascularization (S1 Fig) and muscle survival, demonstrated by comparison with intact muscle fibers in control limbs, was also seen in shPHD-2 versus shScr treated (mean surviving muscle/HPF of 59.9 ± 6.1% versus 33.3 ± 8.8%, *p<0.05) (Fig 4d and 4e). Together, these data support a pro-vascular effect of shPHD-2 application following acute ischemic insult, which promotes tissue survival.

## Discussion

Modulating HIF-1 signaling represents an attractive approach for the treatment of ischemic tissue, with applications ranging from chronic wounds to infarcted myocardium. Recently, animal models genetically modified to enhance HIF-1 signaling have demonstrated improved neovascularization in the setting of ischemia. Specifically, Cre-mediated excision of PHD-2 in the epidermis and dermis resulted in increased HIF-1α stabilization and enhanced wound healing and survival of ischemic tissue [15]. Despite the apparent benefits of decreasing PHD-2 expression, a clinically safe and localized method of suppression is needed before therapeutic regimens targeting this gene can be considered.

Gene therapy allows for a specific and sustained silencing of target genes, but a delivery vector is necessary. Viral vectors, though efficient in transducing many types of cells, are associated with multiple drawbacks. Though recent advances have been made in reducing their immunogenicity [21], our understanding of the host response remains limited [22] and T-cell mediated and humoral immune responses can still be elicited even against less immunogenic viral vectors [23]. Furthermore, due to the potential for insertional mutagenesis, there is an associated risk of cancer [23]. Plasmids, consisting of circular, double-stranded DNA (dsDNA), are one of the simplest vectors for gene therapy, easy to construct, amenable to large volume production [24], and unlike viral vectors, there is negligible immunogenicity associated with plasmids and essentially no risk of oncogenesis as genomic integration is extremely unlikely. Additionally, plasmids can be engineered with large segments of DNA and are stable at room temperature. These attributes make plasmid delivery for gene therapy an attractive alternative.

Here, we utilized a plasmid vector to negatively regulate PHD-2 expression and increase levels of HIF-1α, leading to elevated expression of angiogenic factors SDF-1 and VEGF in murine fibroblasts, consistent with previous studies [15]. Of note, Lijkwan et al. observed similar increases in limb perfusion and vascular density following suppression of PHD-2 [25]. In addition to confirming their findings, we found preservation of digit tips, improved muscle retention, and increased wound healing with our shRNA approach. Zhang et al. also found that negative regulation of PHD-2 improves wound healing, albeit in the setting of fibroblasts implanted into diabetic wounds [26]. In contrast, our study demonstrated beneficial effects on diabetic wound healing with the injection of shPHD-2 alone. This represents a more translational strategy than a cell-based therapy, and obviates any potential risk of fibrosis that injection of fibroblasts may pose.

These findings thus define a functional benefit upon delivery of this therapeutic vector and suggest improved clinical outcomes and quality of life for ischemic limb patients upon translation of this technology. Collectively, our data suggest this technology may have potential applications in other conditions associated with impaired blood supply. Furthermore, prophylactic use of this plasmid in poorly perfused tissue may prevent the sequelae of ischemia, though further studies specifically assessing this potential are needed before any conclusions can be made. Interestingly, Huang et al. found that a simultaneous knockdown of PHD-2 and factor inhibiting HIF-1 (FIH) led to enhanced angiogenesis and stem cell recruitment in ischemic myocardium [27]. This combinatorial strategy might further enhance revascularization in the setting of ischemic limbs and wound healing, and would be worth exploration. Alternatively, PHD-3 may represent another promising candidate for manipulation in the setting of wound healing and ischemia: Loinard et al. found treatment with shPHD-3 to be slightly superior to shPHD-2 in promoting ischemic limb revascularization [28].

The ability to selectively modulate the expression of genes and regulate important biological processes represents an exciting therapeutic platform, with applications extending beyond neovascularization. Fibrotic disease, for example, could potentially be addressed by targeting the focal adhesion kinase (FAK) gene, recently identified as a critical regulator of stress induced fibrosis [29]. As our understanding of regulatory gene networks expands, new targets for plasmid-based therapy will emerge.

## Supporting Information

S1 Fig Local Injection of shPHD-2 Improves Neovascularization Following Ischemic Injury. (a,b) CD31 staining revealed enhanced vascular density in ischemic limb muscle treated with shPHD-2 versus shScr (**p<0.01). Scale bar = 100μm.
(TIF)

S1 Table Primer sequences for genes evaluated with qRT-PCR. (PPTX)
