# Supplementary figures and images for: Short Hairpin RNA Silencing of PHD-2 Improves Neovascularization and Functional Outcomes in Diabetic Wounds and Ischemic Limbs
Source: PLoS One. 2016 Mar 11;11(3):e0150927. doi: 10.1371/journal.pone.0150927 (PMC4788284; doi:10.1371/journal.pone.0150927)

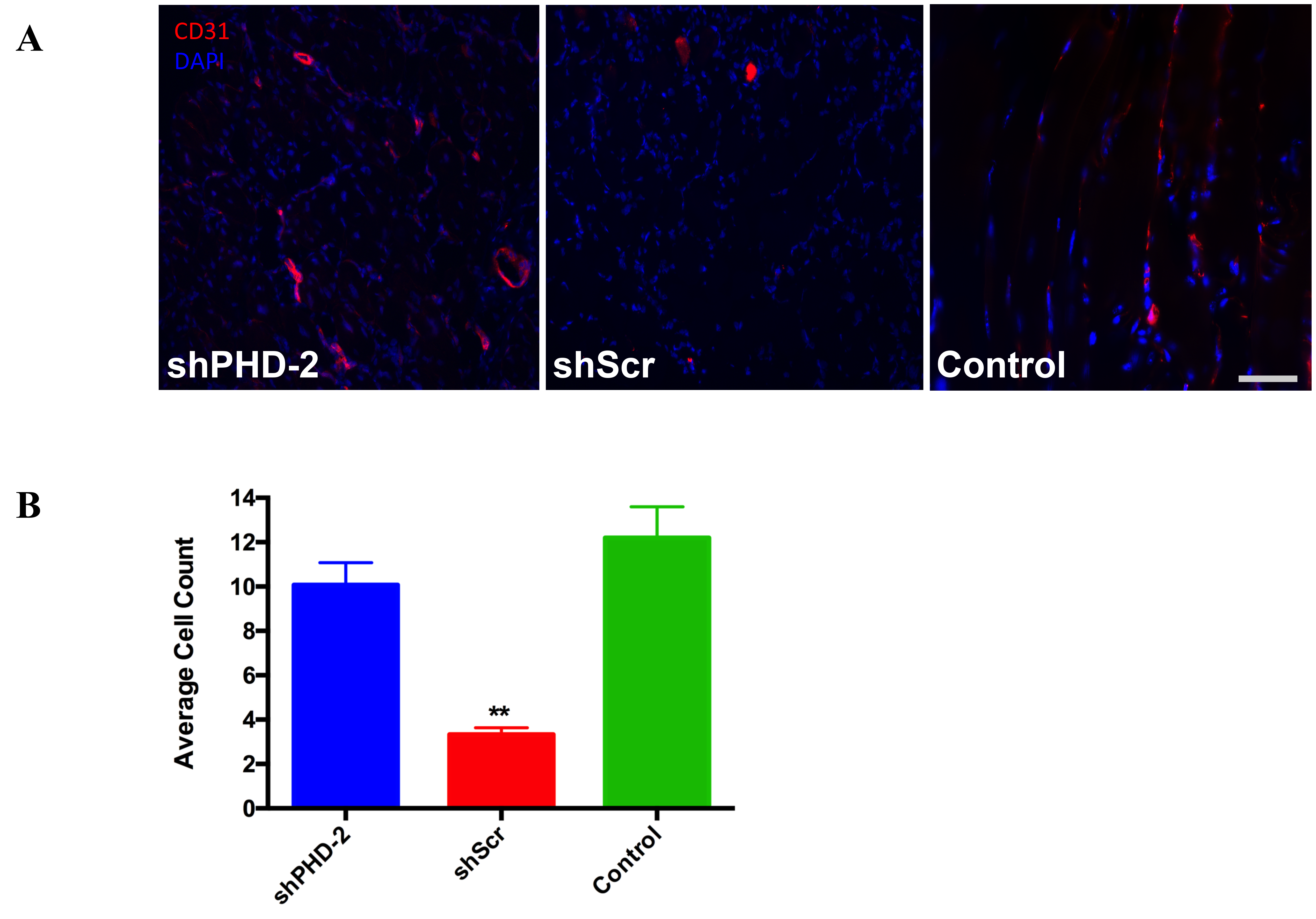

Supplement: S1 Fig — (a,b) CD31 staining revealed enhanced vascular density in ischemic limb muscle treated with shPHD-2 versus shScr (**p<0.01). Scale bar = 100μm. (TIF) [file pone.0150927.s001.tif]

## Slide 1
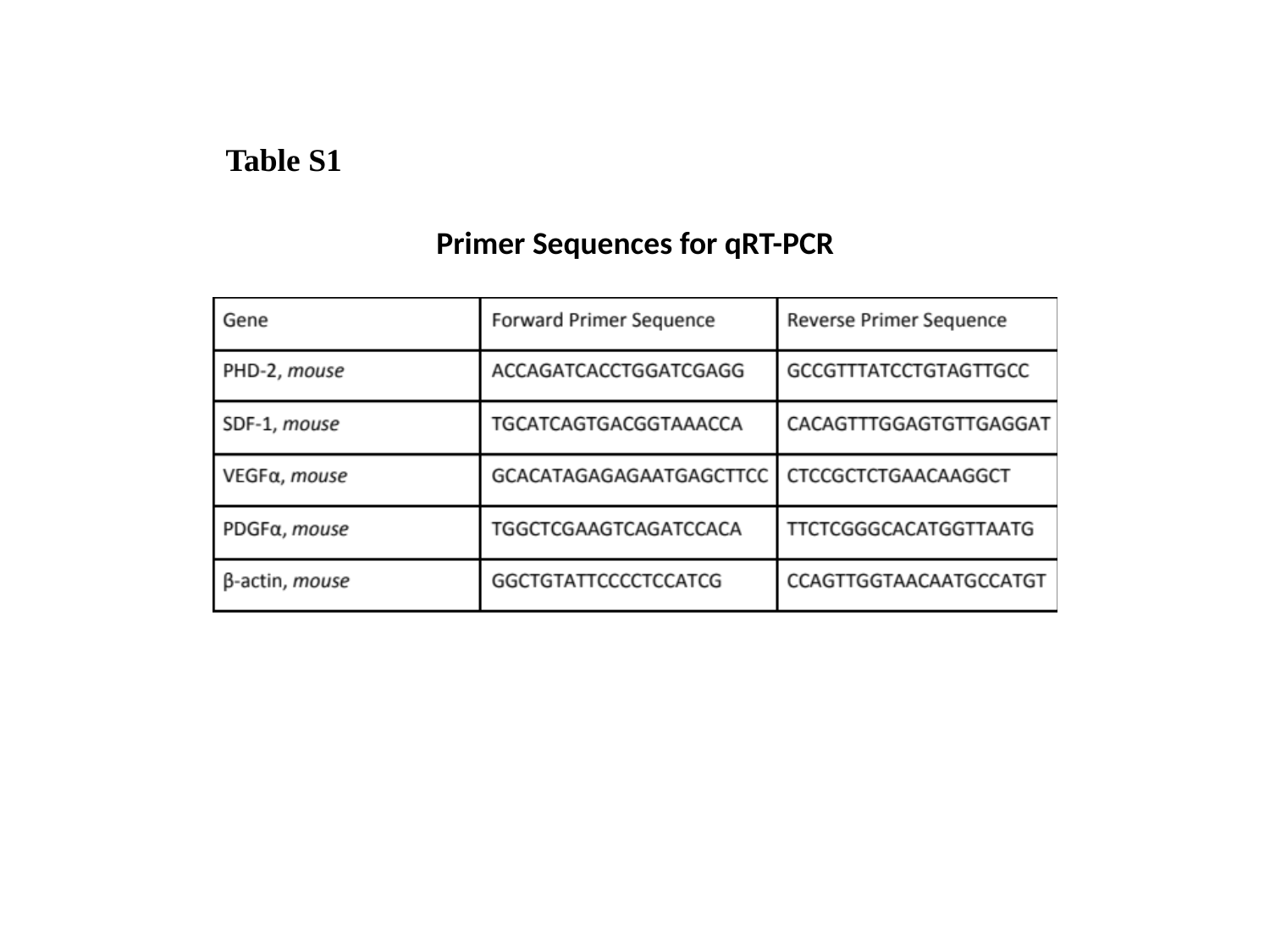

Table S1
Primer Sequences for qRT-PCR

Supplement: S1 Table — (PPTX) [file pone.0150927.s002.pptx]
